# Determinants of pediatric urolithiasis hospitalizations in endemic and non-endemic regions of Mexico

**DOI:** 10.3389/fruro.2026.1704853

**Published:** 2026-05-05

**Authors:** Xally Camila Cahuantzi-Flores, Daniel Flores-Ocotzi, Juan Pablo Flores-Tapia, Rosa Esther Moo-Puc, Edgar Villarreal-Jimenez, Antonio Esqueda-Mendoza, Salvador Gomez-Carro, Nina Mendez-Dominguez

**Affiliations:** 1 Faculty of Medicine, Popular University of the State of Tlaxcala, Tlaxcala, Mexico; 2 Hospital Regional de Alta Especialidad de la Peninsula de Yucatan, Servicios de salud del Instituto Mexicano del Seguro Social para el Bienestar (IMSS-Bienestar), Merida, Mexico; 3 Secretaría de Ciencia, Humanidades, Tecnología e Innovación, Mexico City, Mexico; 4 Hospital General Agustin O´Horan, Servicios de salud del Instituto Mexicano del Seguro Social para el Bienestar (IMSS-Bienestar), Merida, Mexico

**Keywords:** child, hospitalization, Mexico, socioeconomic factors, urolithiasis

## Abstract

**Background:**

Urolithiasis in pediatric populations is increasing worldwide, with environmental and social determinants playing key roles. In Mexico, Yucatan is an endemic area for urolithiasis in adults. However, pediatric data are scarce.

**Objective:**

This study aimed to describe the clinical and ecological characteristics of pediatric urolithiasis hospitalizations in endemic (Yucatan) and non-endemic areas of Mexico.

**Methods:**

We conducted a retrospective, cross-sectional analysis of national hospital discharge records (2018–2024) for patients <18 years with ICD-10 codes N20.0, N20.1, N20.9, N21.0, and N21.1. The sociodemographic and clinical variables were compared between Yucatan and other states. Non-parametric regression models were used to examine the associations between ecological indicators and hospitalization rates.

**Results:**

A total of 2,238 hospitalizations were identified, with a national rate of 3.28 per 10,000 *versus* 28.03 in Yucatan. Patients in Yucatan were younger (mean = 111 *vs*. 128 months, *p* < 0.001) and more likely to self-report as indigenous (4.9% *vs*. 2.3%, *p* = 0.01). The majority of admissions originated from the emergency department (76% *vs*. 64%, *p* < 0.001). In the adjusted models, poverty was positively associated with hospitalization rates (*β* = 0.06, *p* = 0.032), while water hardness and marginalization lost significance.

**Conclusion:**

Pediatric urolithiasis in Yucatan is associated with younger age, indigenous status, and higher hospitalization rates, highlighting the combined role of environmental and socioeconomic factors. Poverty emerged as a key determinant, underscoring the need for targeted preventive strategies, improved access to safe hydration, and culturally sensitive interventions in vulnerable populations.

## Introduction

Recent global analyses using the Global Burden of Disease (GBD) 2021 database indicate that pediatric urinary stone disease represents an important and growing public health concern worldwide. Between 1990 and 2021, the number of incident cases increased from approximately 1.35 million to more than 1.56 million globally, although the age-standardized incidence rate remained relatively stable. During the same period, the mortality and disability-adjusted life-years (DALYs) decreased substantially, reflecting improvements in diagnosis and treatment, but with persistent inequalities across regions (1). In Mexico, public health discourse has frequently attributed the rising burden of chronic kidney disease (CKD) and terminal renal failure to individual behaviors such as excessive consumption of sugar-sweetened beverages, inadequate water intake, and the high prevalence of overweight and obesity, particularly in the southeast region (2). In Yucatan, these risk factors are indeed widespread and have been linked to diabetes, hypertension, and urolithiasis, which are the leading causes of CKD (2, 3). Previous epidemiological studies have described the Yucatan Peninsula as a high-risk or endemic region for urolithiasis due to environmental conditions such as elevated temperatures, high water mineralization, and dietary patterns characteristic of the region (4). Pediatric urinary stone disease differs from adult disease in several aspects. Children are more likely to present with underlying metabolic abnormalities, congenital urinary tract anomalies, or genetic predisposition, highlighting the importance of early evaluation and long-term monitoring (5).

However, framing the problem as a matter of individual responsibility risks obscuring the structural determinants that shape these behaviors, including limited access to safe potable water, food environments dominated by ultra-processed products, socioeconomic vulnerability, and gaps in primary care and early detection services (2, 6). Such a “responsibilization” of health outcomes can stigmatize patients, defer systemic interventions, and exacerbate inequities in a region already facing high CKD mortality and limited renal replacement therapy coverage (3, 7). Although continuous exposure to unhealthy habits may explain many adult cases of urolithiasis, pediatric urolithiasis cannot always be explained by the same mechanisms.

The prevalence of renal lithiasis has been increasing in recent decades, associated with changes in lifestyles, eating habits, the increase in metabolic diseases such as obesity and diabetes mellitus, and environmental and climatic factors (4, 8). Globally, it is estimated that between 5% and 15% of the population will develop an episode of renal lithiasis throughout their lives, with a predominance in young adults between 30 and 50 years of age, being more frequent in men than in women (10.6% compared with 7.1%), but with a progressive reduction in the gender gap noted in recent years (9–11). However, in recent years, there has been an increasing trend in incidence in the pediatric population, which represents a new clinical and epidemiological challenge, especially considering that the main complication of urolithiasis is renal failure secondary to nephrolithiasis.

Recurrence is common in urolithiasis. Long-term cohort studies suggest that approximately 30%–50% of patients experience recurrence within 5–10 years after the first episode, with higher rates reported among pediatric patients (12, 13). Associated pathologies could also be a determining factor in the recurrence of patients, the most common being renal dysplasia, cystic fibrosis, inflammatory bowel disease, and type 1 diabetes or diabetes associated with cystic fibrosis (13). Long-term follow-up studies indicate that the recurrence rates of kidney stones in children range from approximately 12% to 56% within 10 years, underscoring the importance of metabolic evaluation and preventive strategies (5). Therefore, the recurrence of calculi not only complicates the evolution of each patient but also represents a cost for health institutions.

Renal lithiasis remains a persistent global health problem, with increasing incidence particularly in developing countries. In children, the clinical presentation may be atypical and have an increased risk of recurrence. In pediatric patients, the presence of calculi is related to diets of oxalates, animal proteins, and foods rich in calcium and vitamin C, which provide the ideal biochemical environment for the formation of crystals (3). Infants and younger children often present with nonspecific symptoms such as irritability or urinary tract infection, whereas older children more frequently present with flank pain, hematuria, or dysuria (5).

In Yucatan, various studies suggest a progressive increase in the incidence of urolithiasis in individuals over 18 years of age, with significant differences between states, which are attributable to geographical, environmental, and sociocultural factors (14). Geohydrographic studies carried out in the Maya region have shown a water hardness higher than the limits recommended by international organizations, which, together with high exposure to heat, the high prevalence of obesity in the Yucatecan population, and inadequate water intake, could explain the high prevalence of urinary tract disorders (15, 16).

In addition to this, there is little updated literature that accurately describes the characteristics of pediatric patients hospitalized for renal lithiasis in Mexico, which makes it difficult to generate public policies aimed at the prevention and timely diagnosis of this condition. The need for a local epidemiological approach is imperative. Knowledge of the clinical, demographic, environmental, and social characteristics of the affected patients will allow not only measuring the magnitude of the problem but also identifying the risk factors specific to the region. This information will be essential to the establishment of evidence-based intervention strategies, improving the response capacity of the health system.

The objective of this study was to describe the epidemiological profile of patients under 18 years of age hospitalized for urolithiasis in Mexico.

## Materials and methods

We conducted a retrospective observational study based on a cross-sectional analysis of national hospital discharge records from 2018 to 2024. Hospitalizations for urolithiasis in patients under 18 years old recorded by the General Directorate of Health Information (DGIS) between 2018 and 2024 were analyzed. We included discharges with primary diagnoses coded in ICD-10 Chapter N as follows: N20.0, N20.1, N20.9, N21.0, and N21.1. For geographic comparisons, the state of Yucatan was classified as an endemic area based on its hospitalization rate in the general population; the remaining 31 states were grouped as non-endemic areas. All 32 states of Mexico were included as analytical clusters, allowing ecological comparisons between Yucatan and the remaining states.

### Variables and definitions

Sociodemographic variables included age (in months), sex, body mass index (BMI) percentile, and self-reported indigenous status. Self-reported indigenous status corresponds to the variable recorded in the DGIS administrative database based on whether the patient or caregiver reported belonging to an indigenous ethnic group during hospital registration. Clinical variables included length of hospital stay (in days), admission via the emergency department, a different initial diagnosis (non-urolithiasis at baseline), ICD-10 diagnostic category, and subsequent hospitalization attributable to urolithiasis. Urolithiasis was identified using ICD-10 codes N20.0 (calculus of the kidney), N20.1 (calculus of the ureter), N20.9 (unspecified calculus of the urinary tract), N21.0 (calculus in the bladder), and N21.1 (calculus in the urethra). The pediatric hospitalization rate was calculated using the total number of urolithiasis hospitalizations in <18-year-olds as the numerator and the total hospital discharges in <18-year-olds as the denominator, as reported by the DGIS (17). All of the variables and ICD-10 codes used in the analysis are publicly available in the DGIS hospital discharge database, enabling full reproducibility of the analysis using the same data sources.

For the ecological analysis, the 32 states constituted analytical clusters. State-level indicators included the percentage of the population without social security, households with access to drinking water, and the proportion of residents in urban neighborhoods (National Institute of Statistics and Geography, INEGI) (18); the marginalization index (19); the percentage of population living in poverty (20); and environmental variables including the mean temperature and precipitation over the preceding 5 years (National Meteorological Service, SMN) (21), plus deep-water quality parameters (hardness and magnesium, in milligrams per liter) (22).

The distribution of continuous variables was evaluated using the Shapiro–Wilk test. Given the large sample size, variables approximating normal distribution are reported as the mean ± standard deviation. For between-group comparisons (Yucatan *vs*. non-Yucatan), we used a two-group mean comparison test for unequal variances and *χ*^2^ two-group proportion tests, as appropriate. Associations between the ecological characteristics and the hospitalization rates were examined using adjusted and unadjusted non-parametric regression models, reporting beta coefficients (*β*) and *p*-values. Statistical significance was set at *p* < 0.05. Analyses were performed in Stata 19.

## Results

**Figure 1:**
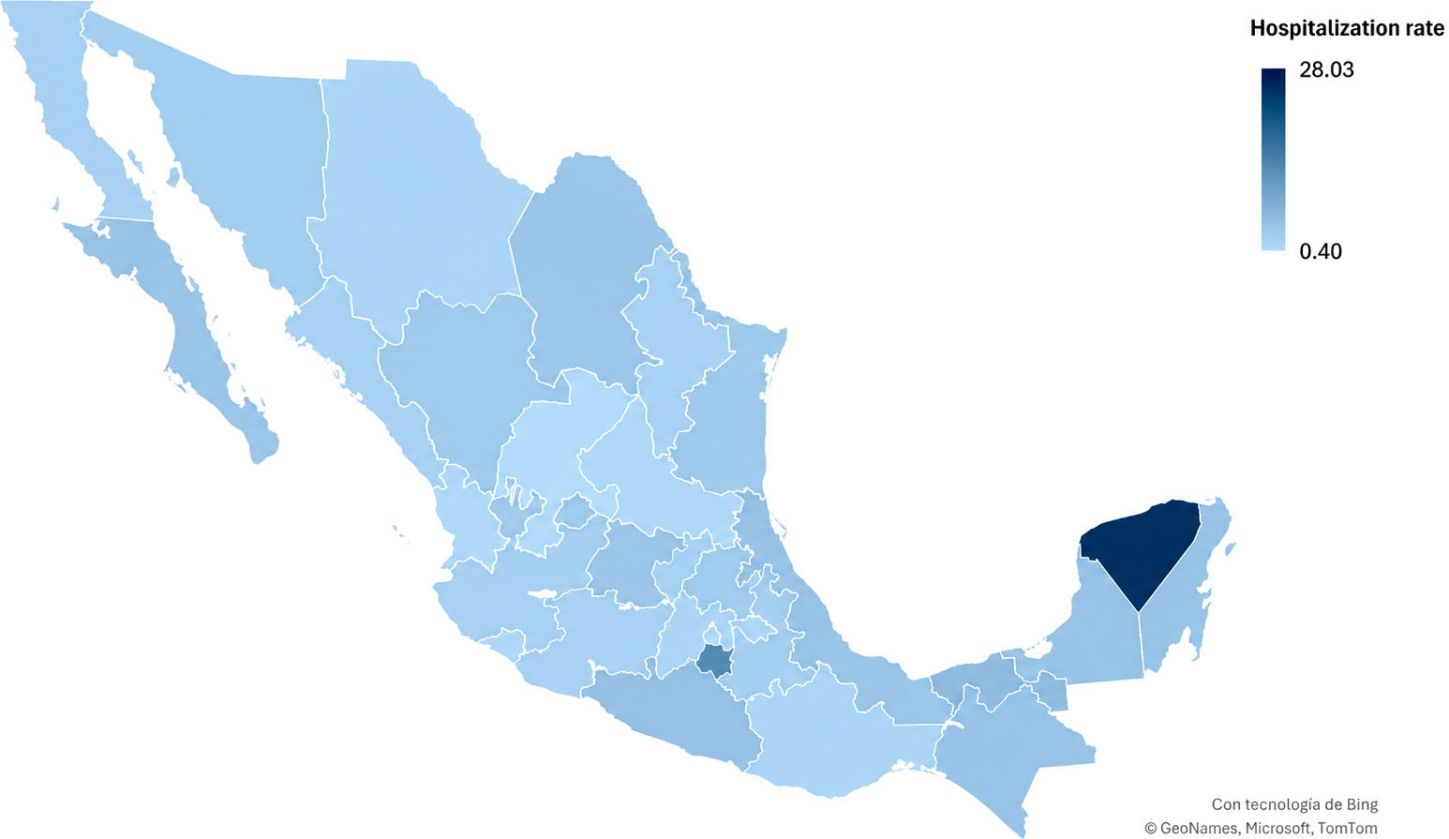
Hospitalization rate per 10,000 hospital discharges among patients <18 years old, Mexico, 2018–2024.

From 2018 to 2024, we identified 2,238 pediatric hospitalizations for urolithiasis. The overall rate for the period was 3.28 per 10,000 pediatric hospital discharges, with the lowest annual rate in 2020 (2.48) and the highest in 2024 (4.11). Yucatan exhibited the highest cumulative rate (28.03). **Table 1** shows the distribution of individual sociodemographic characteristics. At the population level, Yucatan was the state with the highest rate throughout the period with 28.03, as observed in **Figure 1**.

**Table 1:** Description of the population and ecological variables of the 32 clusters.

Variable	Median	25th quartile	75th quartile
% No social security	22.14	18.99	28.60
% Households with access to safe drinking water	97.18	95.28	98.18
% Population in poverty	42.79	28.99	50.84
Marginalization index	19.85	17.95	21.40
Average temperature (°C)	22.85	19.31	25.47
Precipitation (mm)	698.49	477.31	1237.10
% Urban	80.40	69.15	89.01
Water hardness (mg/L)	289.92	190.33	443.23
Magnesium (mg/L)	0.08	0.02	0.15

Regarding patient characteristics, male patients comprised 53.52% (1,198/2,238) and female patients represented 46.47% (1,040/2,238). The mean age was 126.05 ± 66.39 months (10.5 ± 5.5 years), with the highest frequency in the 11- to 15-year group (30.74%). Between-group comparisons showed a lower mean age in Yucatan (*p* < 0.001), a higher proportion of children aged 1–5 years (*p* < 0.001), a lower proportion aged 15–18 years (*p* < 0.001), and a higher percentage with a self-reported indigenous status (*p* = 0.010). Clinically, a higher share of admissions in Yucatan originated from the emergency department (*p* < 0.001), and “kidney stone” was the more frequent diagnostic category (*p* < 0.001). Recurrence was lower in Yucatan, although not statistically significant (**Table 2**).

**Table 2:** Clinical and sociodemographic characteristics of pediatric patients hospitalized for urolithiasis in endemic and non-endemic regions of Mexico, 2018–2024.

Variable	Total: *n* = 2,238 (%)	Yucatan: *n* = 309 (%)	Non-Yucatan: *n* = 1,929 (%)	*P*
Sociodemographic characteristics				
Male, *n* (%)	1,198 (53.52)	164	1,034	0.862
Female, *n* (%)	1,040 (46.47)	145	895	
Age (months), mean ± SD	126.05 ± 66.39	111.44 ± 67.11	128.39 ± 65.99	**0.000**
Self-reported indigenous status, *n* (%)	59 (2.63)	15 (4.93)	44 (2.35)	**0.010**
Age group, *n* (%)				
<1 year	29 (1.29)	9 (2.91)	56 (2.90)	0.992
1–5 years	498 (22.25)	97 (31.39)	401 (20.78)	**0.000**
6–10 years	402 (17.96)	59 (19.09)	343 (17.78)	0.576
11–15 years	682 (30.74)	87 (28.15)	595 (30.84)	0.340
15–18 years	591 (25.40)	57 (18.44)	534 (27.58)	**0.001**
Clinical features				
BMI (percentiles), mean ± SD	74.49 ± 32.66	72.60 ± 32.21	74.80 ± 32.74	0.368
Hospital stay (days), mean ± SD	3.65 ± 6.40	3.97 ± 4.17	3.60 ± 6.69	0.356
Emergency department, *n* (%)	1,470 (65.68)	235 (76.05)	1,235 (64.02)	**0.000**
Different initial diagnosis, *n* (%)	121 (5.40)	10 (3.23)	111 (5.75)	0.069
Kidney stone, *n* (%)	1,312 (58.62)	221 (71.25)	1,091(56.55)	**0.000**
Ureteral stone, *n* (%)	320 (14.29)	37 (11.97)	283 (14.67)	0.209
Unspecified calculus, *n* (%)	315 (14.07)	16 (5.17)	299 (15.50)	**0.000**
Bladder stone, *n* (%)	205 (9.15)	24 (7.76)	181 (9.38)	0.361
Stone in the urethra, *n* (%)	86 (3.84)	11 (3.55)	75 (3.88)	0.781
Recurrence reported, *n* (%)	429 (19.16)	48 (15.53)	381 (19.75)	0.080

The values in bold indicate statistically significant differences.

In the univariable ecological analysis, a higher percentage of households with access to drinking water (*β* = −0.28) and a higher marginalization index (*β* = −0.28) were associated with lower hospitalization rates, while poverty was associated with higher rates (**Table 3**). In the adjusted model, only poverty remained significant (pseudo-*R*^2^ = 20.6%, *β* = 0.06, *p* = 0.032).

**Table 3:** Non-parametric regression of the ecological characteristics associated with pediatric urolithiasis hospitalization rates (state level, *n* = 32).

	Coefficient (*β*)	Standard error	*p*	95% confidence interval	
Medical insurance	0.10	0.05	0.079	−0.01	0.22
Households with access to safe drinking water	−0.28	0.08	**0.003**	−0.46	−0.10
Population in poverty	0.06	0.02	**0.004**	0.02	0.10
Marginalization index	−0.28	0.11	**0.023**	−0.53	−0.04
Average temperature (°C)	0.17	0.10	0.090	−0.02	0.38
Precipitation mm (×10^3^)	2.24	0.49	**0.001**	1.27	3.25
Urbanization	−0.06	0.02	**0.008**	−0.10	−0.01
Water hardness (mg/L) (×10^3^)	1.16	1.77	0.520	−2.47	4.78
Magnesium (mg/L)	−1.79	4.46	0.690	−10.9	7.32

The values in bold indicate statistically significant differences.

## Discussion

We observed a markedly higher pediatric urolithiasis hospitalization rate in Yucatan compared with the rest of Mexico, aligning with prior descriptions in the general population (23). International evidence from hot, arid settings (e.g., Egypt and Turkey) similarly reports higher pediatric urolithiasis prevalence associated with low water intake and increased urinary solute concentration (11, 12), with seasonal peaks during the hottest months. The high proportion of hospitalizations originating from emergency departments, particularly in Yucatan, may reflect delayed diagnosis and limited access to specialized pediatric nephrology or urology services in primary care settings. In regions with high socioeconomic vulnerability, children may reach hospital care only after acute complications such as renal colic, urinary obstruction, or infection occur. Similar patterns have been reported in other low-resource settings, where limited diagnostic capacity and barriers to outpatient care increase emergency presentations.

The mean age at hospitalization in Yucatan (~111 months) mirrors reports from Turkey (12), suggesting earlier manifestation where risk factors cluster. Dietary exposures (including high sodium, animal protein, and sugar-sweetened beverages) are increasingly common in Mexican children and globally (10). In rural Yucatan, cultural norms around unbalanced diets and obesity-related behaviors have been documented, with recent estimates of overweight/obesity of 36.5% in school-aged children and 40.4% in adolescents (24).

A male predominance (53.52%) in the national sample is consistent with reports of a ~2:1 male/female ratio in children under 120 months (13). Among older adolescents, the sex gap narrows. In our study, hospitalized girls were on average older than boys. An increasing incidence among girls has also been observed, potentially linked to urinary tract anatomy, hormonal physiology, and exposure to shared risk factors (25).

In the multivariable ecological models, poverty remained positively associated with hospitalization rates, whereas water hardness and marginalization index lost statistical significance. This pattern suggests that, while environmental factors (e.g., the mineral content of water) may promote stone formation, structural socioeconomic conditions likely mediate hydration practices, diet quality, care-seeking, and access to timely treatment. The attenuation of the environmental and marginalization indicators after adjustment may reflect confounding or collinearity among the ecological variables, underscoring the complex interplay between exposures and social determinants in pediatric urolithiasis. The ecological analysis did not directly compare endemic and non-endemic areas but instead evaluated how state-level environmental and socioeconomic indicators relate to the hospitalization rates across Mexico. Because the ecological indicators were measured at the state level, the regression models assessed associations across all states rather than direct endemic *versus* non-endemic comparisons. Global analyses have demonstrated that the burden of pediatric urinary stone disease varies according to socioeconomic development. Low-sociodemographic index (SDI) regions tend to show increasing incidence rates and slower reductions in disease burden compared with high-SDI regions, where improvements in early detection, preventive strategies, and clinical care have contributed to declining mortality and DALYs (1). The values in bold indicate statistically significant differences. The finding that poverty remained significant after adjustment suggests that socioeconomic conditions may influence exposure to lithogenic environments, hydration practices, and healthcare access, thereby mediating the relationship between environmental risk factors and disease occurrence.

The findings are compatible with literature indicating that poverty and related dietary patterns influence oxidative balance and lithogenic risk (26–28). Environmental factors also contribute to geographic variability. Studies have shown that regions closer to the equator or with higher ambient temperatures tend to experience a higher incidence of urinary stone disease due to increased dehydration and concentrated urine, although this relationship interacts with water quality, dietary patterns, and health system capacity (1). While the BMI percentiles did not differ between endemic and non-endemic areas, the results may serve to motivate further studies of specific dietary patterns in vulnerable populations.

Finally, variables such as indigenous status and BMI percentiles were obtained from administrative records and could not be independently validated. Nevertheless, previous clinical studies have reported that metabolic abnormalities such as hypocitraturia, hypercalciuria, and hyperoxaluria are common among children with urinary stone disease and contribute to stone formation and recurrence (5). Despite these limitations, the use of nationwide data and the comparison between endemic and non-endemic regions provide valuable epidemiological insights.

Narratives that assign responsibility exclusively to individual behaviors are unlikely to reduce pediatric burden in Yucatan. Addressing upstream determinants such as safe and affordable drinking water; regulation of sugar-sweetened beverages and ultra-processed foods, as well as subsidies for healthier options; alongside reinforced primary care, expanded nephrology coverage (including mobile and telemedicine services), and community-based, culturally tailored education are warranted (2, 7, 29–31).

## Conclusion

Pediatric urolithiasis in Yucatan constitutes a significant epidemiological concern, with hospitalization rates far exceeding national averages. The younger age at admission, the predominance of emergency admissions, and the higher proportion of self-reported indigenous status highlight the need to strengthen prevention and early detection, implement culturally sensitive interventions, and improve equitable access to services in vulnerable communities.

Although recurrence was lower in Yucatan than nationally, the ongoing risk of subsequent hospitalization supports sustained clinical follow-up and health promotion strategies.

Our findings are consistent with global epidemiological evidence showing that the burden of pediatric urinary stone disease is increasingly influenced by socioeconomic and environmental determinants, including hydration patterns, climate exposure, and healthcare accessibility.

### Limitations

Given the retrospective, observational nature of the present study, several limitations and cautions must be acknowledged. The ecological design using state-level clusters raises the risk of ecological fallacy, whereby population-level associations may not apply to individuals. Therefore, no extrapolation to individual situations can be supported with the results from the present study. Moreover, we relied on secondary administrative data, which may include coding errors or underreporting. Ethnicity (self-reported indigenous status) data and BMI percentiles were obtained from administrative databases and could not be independently validated. Lastly, no biochemical confirmation (such as urine composition or stone analysis) was available, limiting etiological stone composition interpretation. Despite these limitations, the large national sample and the comparative endemic *vs*. non-endemic analysis strengthen the reliability of the findings.

## Data Availability

Publicly available datasets were analyzed in this study. This data can be found here: https://datos.gob.mx/.
